# Linking GABAergic and Dopaminergic Neurotransmission: Effects on Neural Activity During Human Speech Control

**DOI:** 10.1002/hbm.70531

**Published:** 2026-04-17

**Authors:** Giovanni Battistella, Kristina Simonyan

**Affiliations:** ^1^ Department of Otolaryngology‐Head & Neck Surgery Harvard Medical School and Massachusetts Eye and Ear Boston Massachusetts USA; ^2^ Department of Neurology Massachusetts General Hospital Boston Massachusetts USA

## Abstract

Decades of research were instrumental in identifying the brain function controlling speech production. However, our understanding of the regulation of neuronal excitability via neurotransmission during speaking remains scant as the role of the inhibitory GABAergic system in controlling speech production is unknown. Using PET with [^11^C]flumazenil radioligand combined with PET with [^11^C]raclopride and functional MRI in healthy humans, we investigated the GABAergic neurotransmission and its relationship with dopaminergic function and brain activity during speaking and at the resting state. We demonstrate significant associations between neural activity and GABA_A_ receptor binding during speaking, with positive associations found in the inferior and superior parietal cortices, inferior frontal gyrus, supplementary motor area, superior temporal gyrus, putamen, and negative associations identified in the left inferior and middle frontal gyri. Neural activity was related to the interaction between the GABAergic neurotransmission and nigrostriatal dopamine release in the left associative caudate nucleus and sensorimotor putamen. Conversely, significant correlations between GABAergic neurotransmission and resting‐state activity were limited to the primary visual cortex and the cerebellar lobule VI. These data provide the first direct evidence of the specific interactions of GABAergic and dopaminergic transmission with neural activity controlling the production of speech in healthy humans. Our findings suggest that GABAergic modulation of brain activity is exerted at different stages of speech control, from auditory perception to motor production, whereas dopaminergic function is important for maintaining the balance between excitation and inhibition within the speech motor circuitry.

## Graphical abstract

A multimodal neuroimaging approach using PET with [^11^C]flumazenil radioligand combined with PET with [^11^C]raclopride and functional MRI in healthy humans identified the role of GABAergic neurotransmission and its relationship with dopaminergic function and brain activity during real‐life speaking.

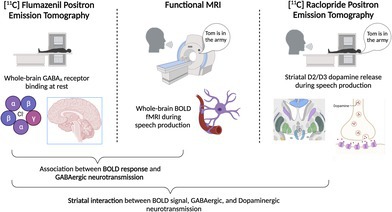

## 1 Introduction

Speech is a unique human behavior that is hierarchically controlled by multiple neural networks associated with sound perception, semantic processing, memory encoding, preparation, and execution of vocal motor commands (Price 2012; Simonyan and Horwitz 2011; Tourville and Guenther 2011). Prefrontal, temporal, and parietal regions represent key nodes of several networks involved in speech‐related cognitive domains. Syntactic processes and auditory‐to‐motor mapping run through a pathway that involves the inferior frontal gyrus and the inferior parietal lobule, while semantic composition and combinatorial processes involve connections from the frontal operculum and inferior frontal gyrus to the middle and superior temporal regions (Bolhuis et al. 2014; Ekert et al. 2021; Friederici 2020; Friederici et al. 2017; Graessner et al. 2021). Speech production constitutes the final stage for encoding articulatory programs into the motor output that relies on fine control coordinated by the speech motor cortex through its widespread organization of cortical and subcortical projection systems (Houde and Nagarajan 2011; Simonyan et al. 2016; Simonyan and Horwitz 2011; Valeriani and Simonyan 2021).

Within the speech production network, neural information transfer relies on rapid neurotransmitter function, as revealed by recent studies investigating dopaminergic neurotransmission during speaking. Specifically, it has been shown that left‐lateralized nigro‐striatal phasic dopamine release directly influences hemispheric lateralization of the functional but not structural neural network during speech production (Fuertinger et al. 2018; Furtinger et al. 2014). In the basal ganglia, endogenously released dopamine during speaking is coupled with neural activity in the associative striatum (Simonyan, Herscovitch, and Horwitz 2013), modulating both direct and indirect basal ganglia pathways (Simonyan et al. 2017).

The dopaminergic neuromodulation during motor actions, including speaking, is performed through its interaction with other neurotransmitters, among which the inhibitory gamma‐aminobutyric acid (GABA) plays a pivotal role (Hoerbelt et al. 2015). Experimental studies have suggested that the maturation of inhibitory GABAergic circuits determines the onset of critical periods in the development and acquisition of language (Werker and Hensch 2015). Multimodal analysis of GABAergic function and resting‐state functional connectivity was able to predict oral reading behaviors, suggesting the effects of inhibitory neurotransmission on the network‐level brain organization (Krishnamurthy et al. 2019). Clinically, GABAergic agents are known to modulate abnormal speech production across various neurological disorders, such as stuttering (Maguire et al. 2010), laryngeal dystonia (De Andrade and Bertolucci 1985; Rumbach et al. 2017; Simonyan and Frucht 2013; Vasilenko et al. 1995), and cerebral palsy (Leary et al. 2006). Furthermore, investigations of the effects of noninvasive neurostimulation techniques on speech and language therapy outcomes in patients with primary progressive aphasia showed that short‐ and long‐term improvements in language scores poststimulation are associated with decreased GABA levels (Harris et al. 2019).

However, unlike studies of dopaminergic function that defined the organization of the dopaminergic neuromodulatory control of speech production in both healthy and diseased populations, our current knowledge of GABAergic neurotransmission during normal speech production is only indirect, derived from the above‐discussed studies of GABAergic alterations in patients with neurological speech disorders. That is, the fundamental understanding of how GABA influences neural activity and interacts with other neurotransmitters for speech control remains unknown. Gaining this knowledge would likely prove beneficial not only for basic research on neurotransmission and speech production but also for informing the development of novel treatments for patients with speech disorders, whose pathophysiology includes GABAergic alterations.

**FIGURE 1:**
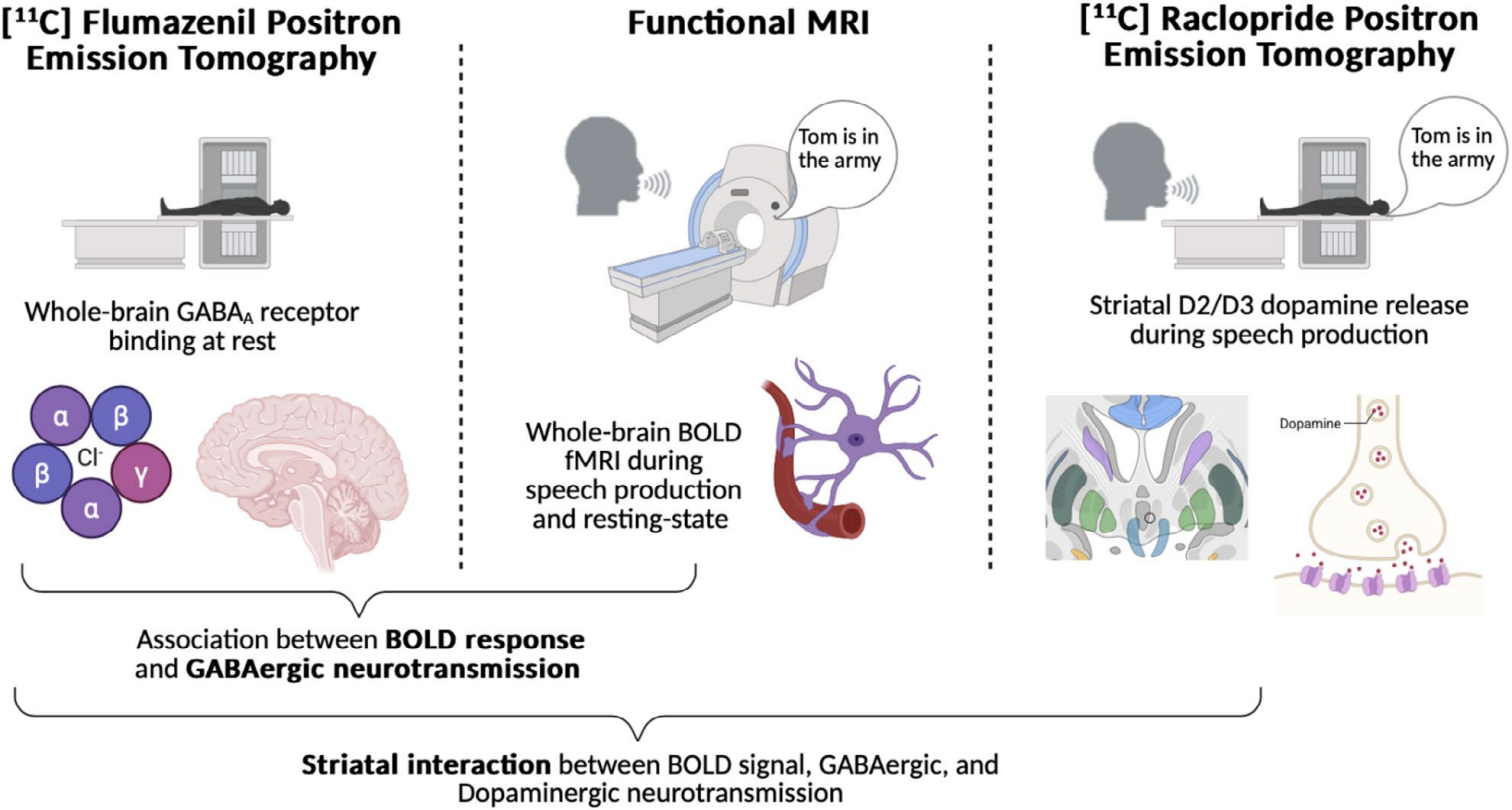
Schematic representation of the experimental design, including [^11^C]flumazenil PET to quantify whole‐brain GABA_A_ receptor binding at rest, task‐based fMRI to investigate whole‐brain BOLD signal during speech production, and [^11^C]raclopride PET to assess striatal dopamine release during speech production. The relationship between GABA_A_ binding potential and BOLD signal and the influence of dopaminergic neurotransmission on the correlation between GABA_A_ receptor binding and BOLD signal during speech production were assessed.

In this multimodal PET‐fMRI study, we investigated (1) the GABAergic neurotransmission by mapping whole‐brain GABA_A_ receptor distribution using [^11^C]flumazenil tracer; (2) the associations of GABA_A_ receptor binding with functional brain activity during resting state and the production of meaningful English sentences with a correct grammatical and lexical structure, and (3) the relationship between GABA_A_ receptor binding and phasic nigro‐striatal dopamine release, measured as the displacement of [^11^C]raclopride tracer bound to striatal D_2_/D_3_ dopamine receptors during production of the same sentences (Figure 1). We hypothesized that GABAergic activity via GABA_A_ receptors is widespread and linked to dopaminergic neuromodulation within the neural network controlling speech production.

## 2 Methods

### 2.1 Subjects

Twenty healthy, monolingual, native English‐speaking, right‐handed volunteers (age 53.2 ± 10.1 years, mean ± SD, 13 females/7 males) participated in the study. None of the subjects had any past or present history of neurological, psychiatric, voice, speech, or language problems based on their medical history, physical examination, and fiberoptic nasolaryngoscopy. The clinical neuroradiological evaluation found normal brain structure without gross abnormalities. All subjects completed two PET studies, one with [^11^C]flumazenil (FMZ) radioligand and the second with [^11^C]raclopride (RAC) radioligand, and two fMRI studies, one during the task‐based overt English sentence production and the second at the resting state (rs‐fMRI, Figure 1).

All subjects provided written informed consent before study participation, approved by the Institutional Review Boards of the National Institute of Neurological Disorders and Stroke, Mass General Brigham, and the National Institutes of Health Radiation Safety Committee. Some data from PET‐RAC and fMRI studies have been reported previously (Simonyan, Berman, et al. 2013; Simonyan, Herscovitch, and Horwitz 2013).

### 2.2 Data Acquisition and Processing

All PET data were acquired on a GE Advance PET scanner (GE Medical Systems, Milwaukee, WI) in the late morning or early afternoon (between 11:34 AM and 2:09 PM) to control for possible diurnal variations in neurotransmission. Prior to participation in the PET studies, subjects were asked to abstain from alcohol for 1 week, from caffeine for 24 h, and fast for 3 h. The PET‐FMZ and PET‐RAC scanning sessions were randomized between subjects.

During the *PET‐FMZ study*, all subjects were instructed to lie still in the scanner with their eyes closed in an environment with dimmed lights and reduced ambient noise and not to fall asleep. A comfortably tight thermoplastic mask was molded around the participant's head to minimize possible head movements. An 8‐min transmission scan was obtained using a ^68^Ge source for attenuation correction of emission data. FMZ was then administered as a 1‐min bolus via a catheter placed in the antecubital vein, and the data were acquired for the following 59 min. Accounting for decay, the effectively injected FMZ activity over a 60‐min PET session was 20.13 ± 0.45 mCi. A dynamic PET scan in the 3D scanning mode with septa retracted was initiated at the start of FMZ injection, generating 21 time‐frames of 30 s to 5 min epochs over 60 min (FOV = 148 mm, reconstructed resolution = 6 mm in all directions).

As a first step in PET FMZ data preprocessing, head motion‐induced artifacts were corrected using the registered attenuation correction method. After the reconstruction of emission images with filtered backprojection with no attenuation correction, all emission frames were registered with mutual information to the prime emission images using the FLIRT toolbox of FSL software. The transmission images were registered to the same prime emission image, and the emission frame was reconstructed with filtered backprojection to be used for attenuation correction. The emission image was resliced back to the transmission position, thus correcting for motion. Using AFNI software, individual quality indices were computed for all preprocessed data to check for remaining residual motions. Final motion‐ and decay‐corrected PET FMZ images were averaged over frames 7–11, which coincided with the FMZ peak uptake, using PMOD Technologies software. The averaged PET FMZ image was registered to the individual T1‐weighted MR image (3D magnetization prepared rapid acquisition gradient echo sequence with TI = 450 ms; TE = 3.0 ms; flip angle = 10°; bandwidth = 31.25 mm; FOV = 240 mm; matrix = 256 × 256 mm; 128 contiguous axial slices; slice thickness = 1.2 mm) using Hellinger distance and the two‐pass alignment method.

Using a two‐step simplified reference tissue model (SRTM2) (Wu and Carson 2002), whole‐brain parametric maps of voxelwise FMZ binding potential (FMZ BP_ND_) were computed within a gray matter mask (Klumpers et al. 2012), which was obtained by segmenting individual T1‐weighted images using SPM software. The pons was manually drawn on individual images and used as a reference region devoid of GABA_A_ receptors (Salmi et al. 2008). The FMZ BP_ND_ images were then spatially normalized to AFNI standard Talairach‐Tournoux space and smoothed with a 6‐mm Gaussian filter. To account for potential outliers in FMZ BP signal variance, voxelwise median absolute deviations (MADs) were calculated for each dataset; the subject was considered an outlier if their values were outside the median ±3.5 × MADs range, as described earlier (Simonyan et al. 2017). Two participants were found to be outliers and, therefore, removed from the final statistical analysis. The statistical parametric map of FMZ BP was computed using data from 18 subjects with a one‐way analysis of variance (ANOVA) at voxelwise family‐wise error (FWE) corrected *p* ≤ 0.05.

*PET‐RAC* and *speech‐production fMRI* data were acquired and processed as reported earlier (Simonyan, Herscovitch, and Horwitz 2013). Briefly, a 100‐min dynamic PET scan (27 time‐frames of 30‐s to 5‐min epochs, RAC activity of 19.7 ± 1.4 mCi, FOV = 148 mm, reconstructed resolution = 6 mm in all directions) in the 3D scanning mode with septa retracted was initiated at the start of a 1‐min RAC bolus injection, followed by a 99‐min constant infusion (Watabe et al. 2000). During the PET‐RAC scan, participants were asked to rest for the first 50 min and then listen to the auditory samples of meaningful English sentences with correct grammatical and lexical structure (e.g., “Tom is in the army”, “We are always away”) and repeat them at a convenient conversational speech level for the remaining 50 min. Ten different sentences were recorded from a native English female speaker for this study, pseudorandomized between subjects, and presented one at a time during scanning. Subjects produced different sentences continuously for 4 min and rested for 1 min to avoid boredom.

Following standard preprocessing as described above for PET FMZ data, the individual parametric voxelwise RAC BP_ND_ maps during speaking were calculated using the SRTM based on the radioactivity concentration in the striatum as a region with the highest density of dopamine D_2_/D_3_ receptors and the cerebellum as a region devoid of dopaminergic receptors (Hall et al. 1996). Next, the percent change in RAC BP (ΔBP) due to the tracer displacement by endogenously released dopamine during speech production was calculated in each subject, followed by the normalization of RAC ΔBP maps using the AFNI standard Talairach‐Tournoux template and smoothing with a 6‐mm Gaussian filter. The statistical parametric map of speech‐induced changes in RAC BP was computed using a voxelwise *t*‐test at corrected *p* ≤ 0.05 (K. Simonyan, Herscovitch, and Horwitz 2013).

The *speech‐production fMRI* included the same 10 sentences used during the PET RAC study and silent fixation as an explicit baseline. Functional images were acquired in a separate scanning session on a 3.0 Tesla GE scanner (TE = 30 ms, TR = 10.6 s with 8.6 s for listening and task production and 2 s for image acquisition, FOV = 240 × 240 mm, in‐plane resolution = 3.75 mm, slice thickness = 4.0 mm) using blood oxygen level‐dependent (BOLD) contrast and a sparse‐sampling event‐related design, as described earlier (Simonyan, Herscovitch, and Horwitz 2013). During fMRI scanning, participants were asked to listen to the auditory sample of a sentence delivered through MR‐compatible headphones for 3.6 s and then visually cued with an arrow to repeat the sentence within a 5‐s interval, followed by a 2‐s image acquisition, during which the participants remained still and silent. The experimental conditions (10 sentences and silent fixation) were pseudorandomized between scanning sessions and subjects. Five scanning sessions were acquired with a total of 36 trials per task condition.

The data analysis followed the standard image processing pipeline described earlier (Simonyan, Herscovitch, and Horwitz 2013). Briefly, task‐related responses were analyzed using multiple linear regression convolved with a canonical hemodynamic response function. Baseline drifts were modeled using quadratic polynomials, and motion parameter estimates were used as additional regressors of no interest. Single‐subject statistical parametric maps of brain activity during speech production were then normalized to the AFNI standard Talairach‐Tournoux template. The group statistical parametric map of speech‐related activation was generated using a mixed‐effect design ANOVA at voxelwise FWE‐corrected *p* ≤ 0.05. No subject was removed from the final analysis due to excessive motion, defined as a relative displacement across volumes greater than the voxel size.

The *resting‐state* fMRI was acquired in the same scanning session as speech‐production fMRI using an echo‐planar imaging pulse sequence (TE = 30 ms, TR = 2 s, 150 contiguous volumes, flip angle = 90°, 33 slices with 3.75‐mm in‐plane resolution, slice thickness = 4 mm). Physiological recordings included measurements of respiration volume and heart rhythm. Data analysis was conducted using AFNI software. Preprocessing included physiological noise removal using the ANATICOIR and RETROCOIR techniques, registration to the AFNI standard Talairach‐Tournoux space, smoothing, and regression of white and gray matter signals, as described earlier (Fuertinger et al. 2015). Single‐subject statistical maps were generated by calculating the correlation coefficient between the average time course of the seed ROI of the whole‐brain gray matter mask and the time course of all other voxels in the gray matter. Single‐subject statistical maps were converted to *z*‐scores by Fisher's r‐to‐*z* transformation. The group statistical parametric map was computed using a one‐sample *t*‐test at voxelwise FWE‐corrected *p* ≤ 0.05.

### 2.3 Multimodal Statistical Analysis

Individual FMZ BP_ND_ and RAC ΔBP maps were volume‐registered and resampled to match the same grid spacing and orientation of the corresponding speech‐related and resting‐state BOLD activity maps in the AFNI standard space. The quality of intermodality alignment was examined visually. To investigate the relationship between GABAergic function and neural activity during speaking and at resting state, voxelwise Spearman's rank order correlation coefficients were calculated between FMZ BP_ND_ and BOLD activity within the gray matter at *R*_*S*_ ≥ 0.5 and voxelwise‐corrected *p* ≤ 0.01 to account for multiple comparisons. To investigate the relationship between GABAergic function, striatal dopamine neurotransmission, and neural activity during speech production and the resting state, we computed voxelwise partial correlations of BOLD percent signal change during speaking or resting and RAC ΔBP or RAC BP, respectively, adjusting for FMZ BP_ND_, within the gray matter. The statistical significance was set at *R*_*S*_ ≥ 0.5 and voxelwise‐corrected *p* ≤ 0.01.

## 3 Results

**FIGURE 2:**
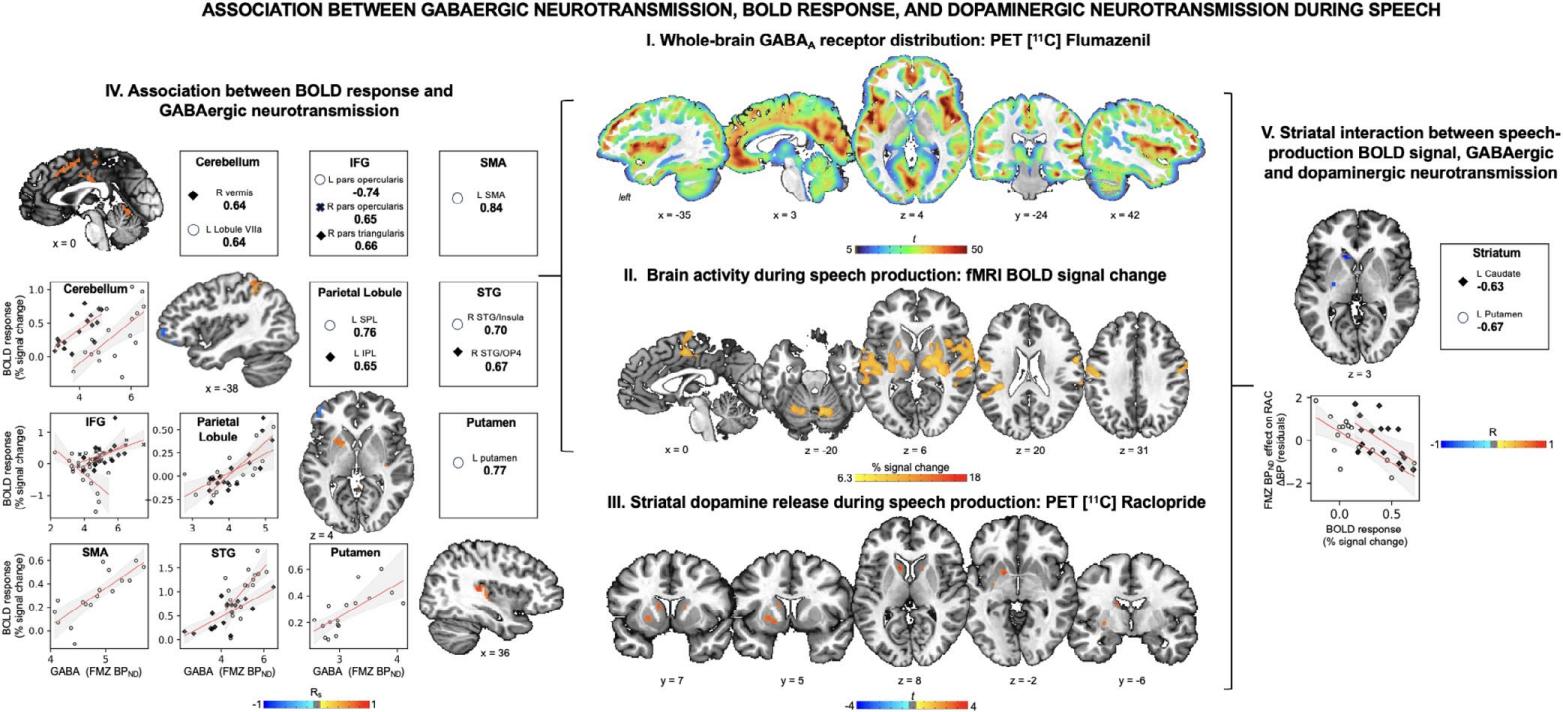
(I) Whole‐brain GABA_A_ receptor distribution measured with [^11^C]flumazenil PET. The color bar represents the *t*‐score of [^11^C]flumazenil binding potential. (II) Whole‐brain BOLD signal during speech production measured as a percentage of the BOLD signal change contrasting rest and speech production conditions. The color bar represents the *t*‐score of the percentage of signal change. (III) Striatal dopamine release during speech production measured with [^11^C]raclopride PET, as reported previously (Simonyan, Herscovitch, and Horwitz 2013). The color bar represents the *t*‐score. (IV) Voxelwise inferential statistics and scatter plots of significant correlation between GABA_A_ binding potential and BOLD signal during overt repetition of meaningful English sentences. The color bar represents Spearman's rank order correlation coefficients. The trend line is in red, and the confidence interval is in gray. (V) Striatal interaction between BOLD signal, GABAergic, and dopaminergic neurotransmission. The color bar represents the partial Pearson's correlation coefficients. The trendline is in red, and the confidence interval is in gray.

**FIGURE 3:**
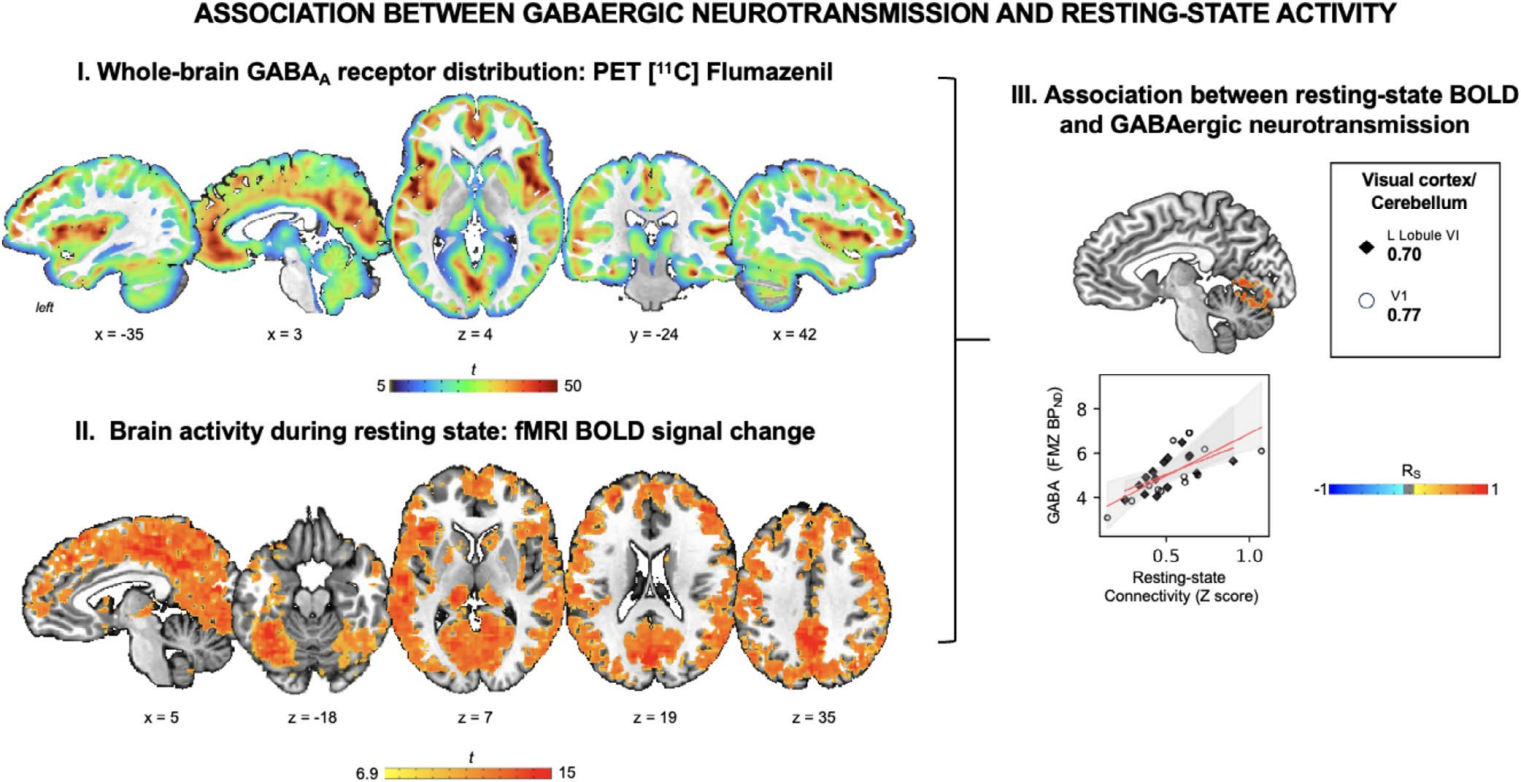
(I) Whole‐brain GABA_A_ receptor distribution measured with [^11^C]flumazenil PET (given here as a reference, same as in Figure 2I). The color bar represents the *t*‐score of [^11^C]flumazenil binding potential. (II) Whole‐brain resting‐state connectivity measured by calculating the significant correlation between the average time course from the whole‐brain gray matter mask and the time course of all other voxels in the brain. The color bar represents the *t*‐score of the percentage of signal change. (III) Voxelwise inferential statistics and scatter plots of significant correlation between GABA_A_ binding potentials and resting‐state activity. The color bar represents Spearman's rank order correlation coefficients. The trendline is in red, and the confidence interval is in gray.

The PET FMZ analysis showed a widespread pattern of statistically significant radioligand binding to GABA_A_ receptors (FWE‐corrected *p* ≤ 0.05, Figures 2I and 3I, Table 1I). Laterally, regions of the highest GABA_A_ receptor binding were identified in the right sensorimotor cortex (BP_ND_ = 4.20 ± 0.37), superior parietal lobule (BP_ND_ = 4.18 ± 0.32), and fusiform gyrus (BP_ND_ = 5.13 ± 0.40); the left inferior temporal gyrus (BP_ND_ = 6.23 ± 0.56) and superior frontal gyrus (BP_ND_ = 5.03 ± 0.41); the bilateral insula (left BP_ND_ = 5.99 ± 0.41; right BP_ND_ = 6.78 ± 0.50), middle frontal gyrus (left BP_ND_ = 5.76 ± 0.43 and BP_ND_ = 5.39 ± 0.44; right BP_ND_ = 5.48 ± 0.40), inferior frontal gyrus (left BP_ND_ = 6.07 ± 0.42; right BP_ND_ = 5.77 ± 0.43), and middle temporal gyrus (left BP_ND_ = 6.24 ± 0.52; right BP_ND_ = 6.11 ± 0.54). The cluster in the left insula extended to the left superior temporal gyrus, parietal operculum, and putamen. The cluster in the right sensorimotor cortex extended to the right inferior parietal lobule. The clusters in the inferior frontal gyrus showed the highest FMZ radioligand uptake in the left pars triangularis and the right pars opercularis. Medially, the highest uptake was found in the left supplementary motor area (SMA, BP_ND_ = 6.86 ± 0.45), extending to the anterior and posterior cingulate cortex and lingual gyrus.

**TABLE 1:** GABA_A_ receptor distribution, brain activity, and dopaminergic transmission during speaking.

I. GABA_A_ receptor distribution			
Anatomical reference	Cluster peak FMZ BP_ND_	Cluster peak coordinates *x*, *y*, *z*	Cluster peak *t‐*value
R sensorimotor cortex extending to inferior parietal lobule	4.2 ± 0.4	46, −19, 47	48.6
R superior parietal lobule	4.2 ± 0.3	23, −62, 53	54.7
R fusiform gyrus	5.1 ± 0.4	33, −30, −18	53.9
L inferior temporal gyrus	6.2 ± 0.6	−46, −50, −14	47
L superior frontal gyrus	5.0 ± 0.4	−6, 54, 28	52.3
R/L insula, extending to superior temporal gyrus, parietal operculum, and putamen	6.8 ± 0.5	36, 14, 4	57.5
	5.9 ± 0.4	−44, 1, 6	61.4
R/L middle frontal gyrus	5.5 ± 0.4	44, 26, 29	57.8
	5.4 ± 0.4	−34, 18, 44	52.3
	5.8 ± 0.4	−34, 39, 29	56.6
R/L inferior frontal gyrus	5.8 ± 0.4	52, 4, 21	57.5
	6.1 ± 0.4	−40, 19, 2	62.0
R/L middle temporal gyrus	6.1 ± 0.5	54, −23, −3	48.4
	6.2 ± 0.5	−59, −39, 3	51.1
L supplementary motor area, extending to anterior/posterior cingulate cortex and lingual gyrus	6.9 ± 0.5	−5, −80, −2	64.1

II. Brain activity during speech production		
Anatomical reference	Cluster peak coordinates *x*, *y*, *z*	Cluster peak *t‐*value
R sensorimotor cortex, extending to superior temporal gyrus, parietal operculum, inferior frontal gyrus, thalamus, putamen, pallidum	51, −11, 34	7.6
L sensorimotor cortex, parietal operculum, superior temporal gyrus	−47, −13, 30	8.9
R supplementary motor area	1, −5, 46	10.9
L putamen, pallidum, thalamus	−25, −9, 2	13.2
R cerebellar lobule V, extending to cerebellar lobule VI	7, −57, −16	14.1
L cerebellar lobule VI	−13, −59, −18	9.7

III. Brain activity during resting state			
Anatomical reference		Cluster peak coordinates *x*, *y*, *z*	Cluster peak *t‐*value
L primary motor cortex, extending to somatosensory cortex		−45, −13, 40	15.5
R premotor, extending to primary motor		53, −1, 36	15.5
L inferior parietal cortex, extending to inferior, middle, and superior temporal gyri		−55, −55, 26	17.2
R inferior parietal cortex, extending to inferior, middle, and superior temporal gyri		53, −63, 26	20.8
L inferior frontal gyrus, extending to insula		−39, 19, 4	23.9
R inferior frontal gyrus, extending to insula		27, 39, −10	17.6
L middle frontal gyrus		−45, 7, 44	15.1
R middle frontal gyrus		39, 41, 18	15.5
L fusiform, extending to right fusiform, bilateral cerebellar lobule VI, visual cortex, precuneus, cuneus, and SMA		−37, −49, −20	22.2
L thalamus		−15, −31, 6	14.7
R thalamus		11, −22, 2	11.7

IV. Striatal dopamine release during speech production			
Anatomical reference	Cluster ΔRAC BP (%)	Cluster peak coordinates *x*, *y*, *z*	Cluster peak *t‐*value
L anterior caudate nucleus	−8.6 ± 12.0	−11, 11, 8	3.3
L posterior caudate nucleus	−9.5 ± 12.7	−15, −5, 20	3.3
L anterior putamen	−6.3 ± 7.9	−15, 3, 2	3.7
L posterior putamen	−6.3 ± 10.0	−27, −7, 0	2.7
R anterior caudate nucleus	−3.9 ± 5.6	15, 7, 12	3.3

*Note:* (IV) Coordinates of striatal dopamine release during speech production measured with [^11^C]raclopride PET were reported previously (Simonyan, Herscovitch, and Horwitz 2013).

Abbreviations: L – left, R – right.

The fMRI during sentence production showed brain activation typical of speaking. That is, active brain regions included the primary sensorimotor and premotor cortex, primary and secondary auditory cortex, parietal operculum, inferior parietal cortex, SMA, basal ganglia, thalamus, and cerebellum (Figure 2II, Table 1II). The fMRI during the resting state showed a widespread pattern, with regions of highest activity identified anteriorly in the left inferior frontal gyrus and insula and posteriorly in the bilateral visual cortex, cerebellar lobule VI, precuneus, and cuneus (Figure 3II, Table 1III).

FMZ BP_ND_ and BOLD signal during speech production showed positive correlations in the left SMA (*Rs* = 0.84, *p* = 2 × 10^−5^), right inferior frontal gyrus (pars opercularis *Rs* = 0.65, *p* = 0.005; pars triangularis *Rs* = 0.66, *p* = 0.004), left parietal lobule (inferior parietal lobule *Rs* = 0.65, *p* = 0.005; superior parietal lobule *Rs* = 0.76, *p* = 4 × 10^−4^), right superior temporal gyrus (cluster extending to the insula *Rs* = 0.70, *p* = 0.002, cluster extending to the operculum *Rs* = 0.67, *p* = 0.003), left putamen (*Rs* = 0.77, *p* = 3 × 10^−3^), and bilateral cerebellum (right vermis *Rs* = 0.64, *p* = 0.006; left lobule VIIa *Rs* = 0.64, *p* = 0.005) (Figure 2IV, Table 2). Brain activity in the cluster covering the left pars orbitalis of the inferior frontal gyrus extending to the middle frontal gyrus showed a negative correlation (*Rs* = −0.74, *p* = 7 × 10^−4^) between FMZ BP_ND_ and neural activity. FMZ BP_ND_ and BOLD signal during resting state showed a positive correlation in the bilateral primary visual cortex and the left cerebellar lobule VI (Figure 3III, Table 2).

**TABLE 2:** Interactions between brain activity and GABAergic and dopaminergic neurotransmission.

Anatomical reference	Correlation coefficient *Rs*	Cluster peak coordinates *x*, *y*, *z*
Correlation between GABA and BOLD response during speech production		
L superior parietal lobule	0.9	−19, −65, 52
L inferior parietal lobule	0.7	−39, −43, 52
R superior temporal gyrus, extending to the insula	0.9	35, −31, 12
R superior temporal gyrus, extending to parietal operculum	0.8	57, −9, 6
R inferior frontal gyrus (pars triangularis)	0.8	39, 27, 6
R inferior frontal gyrus (pars opercularis), extending to insula	0.7	39, 15, 14
L supplementary motor area, extending to middle cingulate gyrus	0.9	−1, −11, 66
R cerebellar vermis (lobules V/VI)	0.8	5, −55, −4
L cerebellar lobule VIIa/Crus I	0.6	−27, −73, −22
L putamen	0.8	−23, 9, 4
L inferior frontal gyrus (pars orbitalis), extending to middle frontal gyrus	−0.8	−45, 39, 2
Interaction of Dopamine × GABA × speech production BOLD		
L caudate nucleus	0.7	−11, 15, 4
L putamen	0.7	−25, −13, 2
Correlation between GABA and BOLD response during resting state		
L calcarine sulcus, extending to right calcarine and left cerebellar lobule VI	0.8	−5, −66, −13

As previously reported (Simonyan, Berman, et al. 2013; Simonyan, Herscovitch, and Horwitz 2013), striatal D_2_/D_3_ dopamine receptors were mapped throughout bilateral putamen and caudate nucleus during the resting state, whereas endogenous dopamine release during speech production was found in both sensorimotor and associative divisions of the dorsal striatum. The latter included the left anterior caudate nucleus (∆RAC BP = −8.6% ± 12.0), left posterior caudate nucleus (∆RAC BP (%) = −9.5 ± 12.7), left anterior putamen (∆RAC BP = −6.3% ± 7.9), left posterior putamen (∆RAC BP = −6.3% ± 10.0), and right anterior caudate nucleus (∆RAC BP = −3.9% ± 5.6, Figure 2III, Table 1IV).

Neural activity during speech production was found to be modulated by the interaction between the GABAergic neurotransmission and striatal dopamine release in the left associative caudate nucleus (*R* = −0.63, *p* = 0.007) and left sensorimotor putamen (*R* = −0.67, *p* = 0.003) (Figure 2V, Table 2). However, there were no statistically significant interactions between GABAergic, striatal D_2_/D_3_ dopamine neurotransmission and resting‐state activity (*R ≤* 0.5, *p* ≥ 0.01).

## 4 Discussion

This is the first study to delineate GABAergic neurotransmission and its interaction with neural activity and dopaminergic neuromodulation during speech production in healthy humans. The whole‐brain mapping of GABA_A_ receptor binding highlights the fine‐grained distribution of FMZ uptake across cortical and subcortical structures, which likely reflects the heterogeneity of GABA_A_ receptor subunits across the brain. The latter is thought to be driven by region‐specific mRNA expression patterns for individual subunits, which in turn influence benzodiazepine binding site density (Nørgaard et al. 2021). Prior research also indicates that the GABA_A_ subunit expression is clustered within regions sharing similar cytoarchitecture (Sequeira Shen et al. 2019), which are more likely to be anatomically connected and functionally linked (Pandya and Sanides 1973; Barbas 2015). In this study, we demonstrate significant associations between GABA and BOLD signals within the speech production network and suggest that these findings are specific to GABAergic neurotransmission for speech control. Particularly, we show that correlations between GABA_A_ receptor binding and neural activity during speaking involve brain regions responsible for the various aspects of speech production, most prominently including preparation to motor execution via the inferior frontal gyrus and SMA, auditory processing via the superior temporal gyrus, sensorimotor integration via the inferior and superior parietal lobules, and goal‐oriented planning and coordination of speech motor action via the putamen and cerebellum. It is notable that these correlations are predominantly positive, indicating a possible role for greater inhibitory activity in modulating higher levels of neural activity during speaking. In contrast, the association between GABA_A_ receptor binding and resting‐state BOLD signal was found in the primary visual cortex extending to the cerebellum, suggesting only minimal influence of GABAergic neurotransmission on baseline intrinsic neural activity. Likewise, prior studies have reported behavior‐specific patterns of GABA/BOLD correlations, such as the relationships in sensorimotor, insular, dorsolateral prefrontal, and anterior cingulate cortices during emotional valence, awareness, and motor learning (Hayes et al. 2013; Wiebking et al. 2014; Lipp et al. 2015; Friedman et al. 2017; King et al. 2020) and in primary and secondary visual cortices during visual stimulation and resting (Muthukumaraswamy et al. 2012; Donahue et al. 2010).

Animal studies have provided further insights into the mechanistic aspects of interactions between GABAergic neurotransmission and neural function. It has been shown that GABAergic neurotransmission reduces overall excitatory activity, leading to lower energy consumption and modulated BOLD response (Chen et al. 2005). GABAergic interneurons are known to densely innervate microvessels and release vasoactive neuromodulators, which contribute to local vascular responses, thus influencing cerebral blood flow (Cauli et al. 2004; Uhlirova et al. 2016; Vaucher et al. 2000; Vazquez et al. 2018). Another potential mechanism involves the balance between GABA and glutamate cycling, in which increased inhibition reduces excitatory drive and metabolic demand (Bonvento et al. 2002; Craven et al. 2024). It is plausible that the identified regional GABA/BOLD associations reflect a change in cerebral blood flow and metabolic rate of oxygen specific to speech motor planning and execution mediated either by glutamatergic excitatory neurons (Attwell et al. 2010; Lauritzen et al. 2012) or by direct influence of inhibitory neurons on BOLD response (Z. Chen et al. 2005). The expected range of inter‐subject variability in the magnitude of the GABA/BOLD correlations may be associated with individual influences of GABA receptor availability on the height and shape of the hemodynamic response function, as shown previously in the visual cortex (Muthukumaraswamy et al. 2012).

The proper fulfillment of motor commands, including speaking, relies not only on GABAergic neurotransmission but also on a delicate balance between inhibitory and excitatory activity, which is fundamental for regulating neuronal firing rate and modulating the direct and indirect basal ganglia pathways (Buzsáki et al. 2007; Fergus and Lee 1997). Animal models suggest that the relationship between GABA and dopamine in the striatum may be driven by the GABA_A_ receptor Beta3 subunit (Pirker et al. 2000; Wisden et al. 1992). This relationship is thought to directly impact the BOLD signal (Donahue et al. 2010; Fergus and Lee 1997; Logothetis et al. 2001) through neurochemical processes driven, in part, by the activation of cortical cholinoceptive GABA interneurons and GABA_A_ receptor‐mediated neurotransmission (Kocharyan et al. 2008). Furthermore, it has been shown that the indirect basal ganglia pathway has greater responsiveness in maintaining the balance between excitation and inhibition and that GABA is more involved in modulating local response to external stimuli than the excitatory neurotransmitter, glutamate (Chen et al. 2019; Gertler et al. 2008). Our findings align with this framework, as the focal interactions between GABA, dopamine, and the BOLD signals in the left caudate nucleus and putamen suggest focal task‐specific striatal dopaminergic modulation of GABAergic neurotransmission during speaking but not the resting state. In addition to the interaction between neurotransmitters in the left sensorimotor division of the putamen, which is related to speech motor output (Simonyan, Herscovitch, and Horwitz 2013), the observed association between GABAergic/dopaminergic function and brain activity in the left associative caudate nucleus may reflect the process by which dopamine tunes inhibition within the striatal circuitry for modulating high‐order cortical cognitive control during speech production. Furthermore, the left‐lateralization of striatal interactions between GABAergic and dopaminergic neurotransmission and BOLD signal during speech production is in line with the previous studies, which showed that endogenous striatal dopamine release influences the left‐hemispheric lateralization of the functional network controlling speech (Fuertinger et al. 2018; Furtinger et al. 2014; Simonyan, Herscovitch, and Horwitz 2013), further underscoring the specificity of our findings to the control of speech production.

The limitations of this study are primarily associated with the inherent limitations of PET imaging. FMZ‐PET is specific only to GABA_A_ receptors, hampering the investigation of the contribution of other GABAergic neurons to functional networks involved in speech production (Boyes and Bolam 2007; Wichmann and DeLong 2003). Similarly, RAC‐PET is sensitive only to striatal dopamine D_2_/D_3_ receptors and does not allow the study of their cortical distribution or dopamine D_1_ receptors, the latter mainly expressed in the direct basal ganglia pathway. Given that PET and fMRI measure neurophysiological processes at different timescales, their temporal mismatch limits the investigation of causal events about dynamic processes of neuromodulation during speech production. Finally, while the current study specifically investigated GABAergic neurotransmission associated with sentence production and the resting state, future studies are warranted to focus on other domains of speech and language control, such as prosody, discourse planning, and auditory feedback.

In summary, this study provides the first experimental evidence for the relationship between GABAergic and dopaminergic neurotransmissions and the organization of neural activity controlling speech production, highlighting the complexity of the central control of human speech.

## Funding

This work was supported by the National Institute on Deafness and Other Communication Disorders (K99/R00DC009629, R01DC011805, P50DC01990).

## Data Availability

The data that support the findings of this study are available on request from the corresponding author. The data are not publicly available due to privacy or ethical restrictions.
